# Supplementary material for: Cell-state transitions and collective cell movement generate an endoderm-like region in gastruloids
Source: eLife. 2022 Apr 11;11:e59371. doi: 10.7554/eLife.59371 (PMC9033300; doi:10.7554/eLife.59371)
Supplement: Supplementary file 1. [file elife-59371-supp1.docx]

**Supplementary File 1 - Statistics**

| Figure | Sample # (analysis) | Replicates | Statistical test | Miscellaneous |
| --- | --- | --- | --- | --- |
| 1B | n = 20 aggregates (control), n = 37 aggregates (Chi+) | N = 2 | Mann-Whitney test with correction | box whisker plot with 25th and 75th quartile; all data points are superimposed. p-value = 3x10^-5^ |
| 1C | Same as 1b | Same as 1b | Mann-Whitney test with correction | box whisker plot with 25th and 75th quartile; all data points are superimposed.  p-value = 7x10^-10^ |
| 1E | n = 7 aggregates (control), n = 7 aggregates (Chi+) | Control: N = 1; Chi+: N = 3 | N/A | Each marker represents a different aggregate |
| 1F | n = 14 aggregates | N = 3 | N/A | thick and dashed lines delineate mean and mean ± SD respectively |
| 1G | n = 11 aggregates | N = 3 | N/A | Same as 1f |
| 2B | n = 3 aggregates | N = 2 | N/A | error bars in histogram represent SD |
| 3B | n = 7 aggregates | N = 2 | N/A | error bars in histogram represent SD |
| 3C | Same as 3b | Same as 3b | N/A | Spatial probability density function computed from pooled data |
| 3E | Islands from n = 6 aggregates | N = 2 | N/A | Normalized T-Bra and Oct4 intensities within/around pooled islands |
| 4A | n = 1 two-photon video | N = 3 | N/A | Velocity vector direction is color-coded. The velocity averaged over the whole aggregate is subtracted to the velocity field at each timepoint. |
| 4B | n = 1 two-photon video | N = 3 | N/A |  |
| 4C | n = 28 junctions at 72 h, n=54 junctions at 96 h | N = 2 | Kolmogorov-Smirnov Test | box-whisker plot of the junction tension ratio with 25th and 75th quartiles; data points are superimposed; P-value = 0.37 |

| Supplements | Sample # | Replicates |
| --- | --- | --- |
| Fig 2 – supp 1 | E-cad/Ph3/Foxa2: n = 5 aggregates; Foxa2/E-cad/T-Bra: n= 3; Sox17/E-cad/T-Bra: n= 13 aggregates; E-cad/Sox17/Sox 2: n=5 aggregates | N = 2; N=2; N = 2; N = 1 |
| Fig 3 – supp 1 | n = 4 (panel A); 7 (B); 5 (C) | N = 1(A); 1(B); 1 (C) |
| Fig 3 – supp 2 | n = 5 | N = 1 |
| Fig 4 – supp 1 | Control: n = 4 aggregates; pulsed: n = 3 aggregates; | N = 2 |
